# Pharmaceutical market access in emerging markets: concepts, components, and future

**DOI:** 10.3402/jmahp.v2.25302

**Published:** 2014-12-01

**Authors:** Anuj Kumar, Karthaveerya Juluru, Phani Kishore Thimmaraju, Jayachandra Reddy, Anand Patil

**Affiliations:** phamax Analytical Resource Pvt. Ltd., Bangalore, India

**Keywords:** pharmaceutical market access, emerging markets, developed markets, stakeholders, market access framework, escalating healthcare cost, pricing and reimbursement

## Abstract

This article intends to consolidate the concepts of pharmaceutical market access and highlight its growing importance in emerging markets.

Market access has gained considerable attention worldwide as countries try to contain their escalating healthcare expenditures amidst the global economic slowdown. This has resulted in governments adopting stricter measures for new product approval. Thus, pharmaceutical companies are finding it increasingly difficult to successfully address the specific challenges posed by various government and regulatory agencies and stakeholders.

There is an increasing need to establish market access functions, especially in emerging markets, where the complex, dynamic healthcare landscape confounds product approval and uptake. Moreover, emerging markets are the engines of growth today, and, thus, performing in these markets is critical for the majority of pharmaceutical companies.

To address the challenges posed by regulatory agencies and diverse stakeholders, a customized market access strategy is the need of the hour. A market access framework with specific tools and tactics will help companies to plan, implement, and monitor stakeholder engagement activities.

Over the last decade, the global pharmaceutical market access landscape has undergone a significant transition and has garnered substantial attention. This is primarily due to the healthcare reforms promulgated by governments across the globe to contain their burgeoning healthcare expenditures (1).

Traditionally, R&D, sales, and marketing have been the predominant drivers of commercial success for pharmaceutical companies (2, 3). This traditional market access approach is very linear and involves engaging with physicians, pharmacies, and regulatory bodies for greater product uptake (4), but it includes only pricing and reimbursement activities. However, we define market access as a process that ensures all appropriate patients have rapid and continued access to the product at the *right* price. This indeed is a broad concept that includes multiple functionalities from a company's commercial, regulatory, supply chain, marketing, medical, and corporate functions. There has already been a recent shift in the makeup of stakeholders to also include patients, payers, and advisory groups due to their increased role in treatment decision making. These changes have forced an evolution in market access to a value-based approach from the traditional price-based perspective. This has made market access an increasingly important area of focus for pharmaceutical companies to achieve success through improved access (3).

Generally, market access is perceived as a function that is confined to pricing and reimbursement activities. However, in reality, it is a multidisciplinary field that includes aspects from various other business functions, such as managing channels, stakeholders, and key opinion leaders (KOLs).

This broad concept of market access is already in the process of implementation in Western markets, with companies establishing dedicated or cross-functional teams that can handle the multiple facets of market access (5). In contrast, the market access setups of pharmaceutical companies targeting the emerging markets (e.g., Latin America; Brazil, Russia, India, and China [BRIC]; the Middle East; etc.) are still nascent and have yet to attain the level of significance they hold in the Western markets.

Of course, the diverse market characteristics and dynamics dictate the market access situations in these emerging markets. Reimbursement does not exist in many markets, and where it does exist, there are several restrictions. Thus, access in emerging markets is largely a function of price, channels, marketing to doctors, and government stakeholders. However, companies working in these emerging markets focus on either individual functions or none at all.

Regardless of the differences from developed markets, the emerging markets are still the growth powerhouses, and so companies are increasingly looking toward them to generate revenue growth. Considering this situation, it is indeed a foregone conclusion that market access will soon become an important aspect of marketing strategy in these markets as well. Of course, companies can apply their learnings from Western markets while developing market access strategies for these markets. In sum, the success of not just a new product but also the organization itself depends heavily on a robust market access strategy that is customized to the challenges of a particular market or country.

## Market access – then and now

The pharmaceutical industry has traditionally relied heavily upon the *push* strategy to ensure their products succeed in the market. The drug approval process was simple: it involved submitting data on efficacy, safety, and tolerability to the regulatory agencies. Once approval was secured, the drug was marketed to the targeted physicians and dispensed by pharmacies. Thus, market access involved engaging with a limited set of stakeholders: physicians, regulatory agencies, and pharmacies.

However, over the years, the market access landscape has evolved primarily due to two factors:

1. Escalating healthcare costs due to a growing prevalence of chronic diseases, increase in the geriatric population, and higher prices of new therapies: As in developed countries like the United States, where the rise in healthcare cost is an integral function of diagnostic and allied costs, the emerging markets also behave in a similar fashion owing to their recent advancements and reforms in healthcare systems.
2. Challenging pricing and reimbursement environment: Increased scrutiny of the claimed product value by healthcare authorities is restricting the pricing and reimbursement space for new products. Reference pricing and generic substitutions are among the already implemented techniques to improve affordability for marketed products (6).

These two factors have resulted in the emergence of a new and diverse set of stakeholders over the years. However, this has increased the complexity of drug access to the market in general, and to patients in particular.

## Emerging stakeholders in market access

The **Payer** is undoubtedly the stakeholder with the highest prominence. The Payer exercises the greatest degree of control over pricing and reimbursement for any new drug (7). There are dedicated health technology assessment agencies that advise payers in reimbursement and formulary decisions. Payers also proactively participate in establishing treatment protocols and have an influence on prescribing behavior of physicians. Payers will continue to dominate the market access scenario and are key to a new product's success on the market (8).

**Patients** today are more aware of treatment modalities and can be expected to demand justification for the price charged for a drug. This is because patients have to bear the additional cost of high-priced drugs through co-payments (in such cases, they become payers as well). In addition, Patients are more concerned about the effectiveness of the drug than earlier, and they are not satisfied with just receiving treatment but also demand a cure. Indeed, the importance of drug effectiveness will further increase if there is no reimbursement or only partial reimbursement.

**Pharmacies** are key stakeholders who could influence drug access by controlling the availability of the product in the retail or out-of-pocket market. In cases of reimbursement, pharmacies could also influence the choice of brand through substitution. Understanding their dispensing behavior and securing the most shelf space are important for product success.

**Advocacy groups** are gradually beginning to exert their influence as stakeholders in market access, especially in niche therapies where the cost of treatment is very high (e.g., in rare diseases). Moreover, they wield considerable influence in healthcare policy shaping and indirectly affect treatment guidelines.

**Physicians and KOLs** have seen some reduction in their importance in the market access value chain over the years. The growing austerity measures have influenced their prescription behavior to a considerable extent. As companies struggle to spend quality time with these important traditional channels, it will be a challenge to effectively engage and explore areas of common interest.

**Government bodies and regulatory agencies** are a complex group of stakeholders that play a vital role in shaping healthcare policy and establishing a framework for pharmaceutical companies to operate within (e.g., setting pricing and reimbursement guidelines). In some countries, they are the payers, and they hold the key to the pharmaceutical market and will continue to exert significant influence. Pharmaceutical companies will need to effectively manage this extremely challenging group of stakeholders to succeed in the market (9).

The decision-making landscape in the healthcare system has become quite complex, with intertwined relationships among various stakeholders. The involvement of different stakeholders in the market access process varies by therapeutic area. Knowing the relevant stakeholders as well as their needs and interdependencies is critical as it greatly determines the success of market access activities.

## Market access scenario in developed markets versus emerging markets

In the developed markets, the market access function has steadily attained importance due to increased awareness of the need for value over existing treatments among regulatory and reimbursement agencies. To deal with this dynamic regulatory environment, pharmaceutical companies have started to establish the market access function as an integral part of the organization. However, only a handful of companies currently have a dedicated market access team with well-defined roles and responsibilities (10, 11). Instead, the majority of pharmaceutical companies currently have a splintered approach, with market access responsibilities being shared among sales, marketing, and regulatory divisions.

In the emerging markets, market access is still not as well structured as in the developed markets. However, the changing market landscape and evolving healthcare policies have led to increased importance of market access functions. Despite this, currently, pharmaceutical companies are focusing on individual components of market access (price, channel, stakeholders, and government agencies), but there is no holistic approach to deal with all components together. Moreover, the healthcare policies and regulatory landscapes in these markets are more complex than in the developed markets. Pharmaceutical companies thus find it difficult to identify the right stakeholders that need to be engaged as part of the drug approval process. Moreover, companies do not have established processes, plans, and talent to circumvent the challenges posed by the various stakeholders in market access. Hence, there is a greater need for a dedicated market access team. Taking note of this, a few pharmaceutical companies have started to establish market access functions (10, 11).

## Barriers to market access and commercial success in emerging markets

Market access involves engaging with all components of a market and with different stakeholders who impact the overall product commercialization process. Thus, customized processes and functions are required to effectively engage these stakeholders. The key challenge is to integrate these processes and functions so as to minimize overlap and duplication, which might result in suboptimal product uptake and wastage of resources. Moreover, market access teams in the emerging markets face several unique challenges (12):

1. Lack of organizational support (e.g., budgetary constraints to develop a new team dedicated to market access)
2. Dearth of resources at both global and local offices
3. Low level of cooperation between different functional teams (e.g., medical, marketing, etc.)
4. Lack of alignment and integration of market access activities among divisions operating in different countries or markets
5. Paucity of data critical to market access function (e.g., data on pricing, reimbursement, tenders, formularies, etc.)
6. Identification of the right stakeholders and effective engagement with them
7. Identification of areas of national importance from a policy-shaping standpoint

## Key success factors for market access function in emerging markets

To succeed in such complex markets, a pharmaceutical company will require a comprehensive market access strategy. This has to be closely aligned with other corporate functions and customized to local challenges. The healthcare landscape in the emerging markets is complex and does not follow a structured drug approval process, as compared to the developed markets. Market access involves various processes and activities for engaging with a diverse set of stakeholders. The following are the key success factors that a company needs to adopt to gain smooth market access:

1. Integrated market access strategies, beginning from the product development stage
2. Developing a culture of *team effort* by facilitating effective collaboration among various business functions (e.g., sales, marketing, regulatory, etc.)
3. Key account management (KAM) or specialized teams dedicated to managing stakeholders
4. Adopting an integrated stakeholder management approach
5. Better understanding of the relationship between market access and stakeholders
6. Effective communication with internal and external stakeholders
7. Establishment of optimal processes, plans, and, most importantly, people

In addition, the market access strategy has to be closely aligned with other corporate functions and implemented through appropriate tactics to ensure product success. A dedicated market access team with a collaborative working dynamic, built through a brand-team culture, will enhance the speed of product uptake and act as a catalyst for organizational growth.

## Market access in future

The current volatility in the global economy is expected to continue into the near future. Moreover, pressure from governments to contain their burgeoning healthcare bills will also continue. Thus, market access is expected to assume greater significance, particularly in the emerging markets. Pharmaceutical companies need to proactively engage with the key stakeholder groups in order to keep up with their potential future needs. This is critical to effective product commercialization since the future success of an organization often hinges on its ability to understand and embrace changes in a dynamic pharmaceutical environment. These changes should not only be confined to organizational structure but also percolate to all business processes. Most importantly, pharmaceutical companies must adopt a *market-access-oriented* organizational mindset.

The stagnant developed markets have forced pharmaceutical companies to focus more on the emerging markets, which are touted as being the next engines of growth. Thus, growth for any pharmaceutical company will depend on its performance in these markets. A customized market access strategy integrated with the right processes and talent can help mitigate these challenges, allowing effective commercialization and increased drug accessibility for patients.
